# Down-regulation of HSPB1 and MGST1 promote ferroptosis and impact immune infiltration in diabetic cardiomyopathy

**DOI:** 10.21203/rs.3.rs-5153598/v1

**Published:** 2024-12-09

**Authors:** Yaoli Xie, Bin Liang, Zhijun Meng, Rui Guo, Caihong Liu, Yi Yuan, Wei Mu, Yajing Wang, Jimin Cao

**Affiliations:** Shanxi Medical University; Shanxi Medical University; Shanxi Medical University; Shanxi Medical University; Shanxi Medical University; Shanxi Medical University; The Fifth Hospital of Shanxi Medical University (Shanxi Provincial People’s Hospital); University of Alabama at Birmingham; Shanxi Medical University

**Keywords:** HSPB1, MGST1, Diabetic cardiomyopathy, ferroptosis, immune infiltration

## Abstract

Diabetic cardiomyopathy (DCM) is a leading cause of death in diabetic patients. Current therapies do not adequately resolve this problem and focus only on the optimal level of blood glucose for patients. Ferroptosis plays an important role in diabetes mellitus and cardiovascular diseases. However, the role of ferroptosis in DCM remains unclear. Differentially expressed ferroptosis-related genes (DE-FRGs) were identified by intersecting GSE26887 dataset and the Ferroptosis Database (FerrDb). The associations between the DE-FRGs and immune cells in DCM, estimated by CIBERSORTx algorithm, were analyzed. Using ow cytometry (FCM) to evaluated the infiltration of immune cells of myocardial tissues. The expression of DE-FRGs, Glutathione peroxidase 4 (GPX4) and Solute carrier family 7 member 11 (SLC7A11) were examined by real-time quantitative PCR and western blotting. 3 DE-FRGs were identified, which are Heat shock protein family B (small) member 1 (HSPB1), Microsomal glutathione S-transferase 1 (MGST1) and solute carrier family 40 member 1 (SLC40A1) respectively, and they were closely linked to immune cells in DCM. In vivo, the levels of CD8 + T cells, B cells and Treg cells were significantly decreased in the DCM group, while the levels of CD4 + T cells, M1 cells, M2 cells and monocytes were increased. Diabetes significantly decreased HSPB1 and MGST1 levels and increased ferroptosis compared to normal group. Furthermore, ferroptosis inhibitor ferrostatin-1 (Fer-1) alleviated high-fat diet (HFD)-induced cadiomyocyte injury and rescued the ferroptosis. This study suggests that ferroptosis related gene HSPB1 and MGST1 are closely related to immune cell infiltration, which may become therapeutic targets for DCM.

## Introduction

Diabetic cardiomyopathy (DCM) is a specific form of cardiomyopathy, promoted by type 2 diabetes mellitus, insulin resistance and associated hyperinsulinaemia, which occurs independently of other heart disease risk factors including hypertension, coronary artery disease (CAD), valvular disease, and so on[1, 2]. The higher risk of cardiovascular disease in people with diabetes is attributed to persistent high glucose status, insulin resistance and long-term metabolic disorders [1, 3]. This specific form of cardiomyopathy has a high rise of morbidity and mortality gains considerable attention in the world. DCM is characterized by abnormal myocardial structure and function[3]. Studies indicated that the main impacts of diabetes on the heart are left ventricular diastolic dysfunction, reduced left ventricular ejection fraction, reduced left ventricular compliance, left ventricular hypertrophy and interstitial fibrosis [4–6].

Many forms of cell death, including necroptosis, apoptosis and pyroptosis, have been verified involved in cardiovascular diseases (CVD) [7]. Ferroptosis is a new type of iron-dependent regulatory cell death, and this type of cell death is non-apoptotic and associates with the accumulation of lipid hydroperoxides [8]. In recent 10 years, a growing number of studies support the opinion that ferroptosis involves in the development of cardiovascular diseases, including myocardial ischemia–reperfusion injury, doxorubicin-induced cardiomyopathy, heart failure and myocardial infarction [7, 8]. Both primary and secondary iron overload can lead to heart disease via oxidative damage [9]. Related studies have shown that additional iron in cardiomyocytes can directly induce ferroptosis through accumulating phospholipid hydroperoxide on the cell membrane [10]. Indeed, oxidative stress has been related to the cardiovascular disease, particularly the antioxidant system of glutathione (GSH)-dependent has been shown to protect against cardiomyopathy [11, 12].

Here, we identified the differentially expressed genes (DEGs) in the hearts of DCM patients and non-DCM patients, and then matched these DEGs to the ferroptosis dataset to acquire differently expressed ferroptosis-related genes (DE-FRGs) to determine the mechanism of DCM development. A protein-protein interaction (PPI) network was constructed to identify key functional modules and hub genes. Furthermore, we performed immune infiltration correlation analyses of DE-FRGs in DCM to obtain the tight associations among DCM, ferroptosis and immune infiltration. We also constructed a transcription factor (TF)-miRNA-DE-FRGs network to predict that DE-FRGs are regulated by microRNA. Finally, we established a mouse model of DCM to detect the immune infiltration in the myocardium by FCM and to verify the expression of key ferroptosis-related genes (FRGs) in DCM of mice by PCR and western blotting. We further confirmed that ferroptosis was involved in the process of DCM through in vivo and in vitro experiments. These findings reveal the role of ferroptosis in DCM and suggest that ferroptosis may become a target of DCM therapy in the future.

## Materials and methods

### Data collection

The GEO (http://www.ncbi.nlm.nih.gov/geo) is a public database, which contains a variety of diseases of microarray and high-throughput sequencing datasets. We recruited 7 patients with DCM and 5 normal control patients from GSE26887 dataset. In addition, the FRGs used in this study were derived from the Ferroptosis Database (FerrDb, http://www.zhounan.org/ferrdb), which is the world’s first database that dedicates to ferroptosis regulators and ferroptosis-disease associations.

## Identification of differently expressed ferroptosis-related genes

The GEO2R (https://www.ncbi.nlm.nih.gov/geo/geo2r/) was used to screen DEGs between the DCM group and the normal group. The screening criterion was adjusted to P < 0.05 and |log2FC|≥1. Heatmap and volcano plots of DEGs from the databases were constructed using R packages and GEO2R. We selected the overlapping DEGs of FRGs as DE-FRGs.

## Analysis of receiver operating characteristic curve

The DE-FRGs in the validation dataset GSE26887 were considered as diagnostic biomarkers for DCM. Based on the expression of DE-FRGs in GSE26887, Graphpad Prism was used to compare the normal group and DCM group. Area under the curve (AUC) of ROC curve analysis was used to evaluate the diagnostic value of DE-FRGs for DCM.

## Construction of PPI Network

We constructed PPI network diagrams by uploads DE-FRGs to GENEMANIA (http://genemania.org/), which can find other genes that are related to a set of input genes, using a very large set of functional association data [13].

## Analysis of immune cell infiltration

We uploaded the expression pro les of GSE26887 to CIBERSORTx (https://cibersortx.stanford.edu/) to analyze immune cell infiltration. The software tool provided an estimation of the abundances of member cell types in a mixed cell population, using gene expression data [14]. First, we used CIBERSORTx to assess the proportion of immune cell infiltration in each sample. Then, we performed Spearman’s rank correlation analysis between immune cells and DE-FRGs.

### Animal model

All animal experiments were performed in adherence to the National Institutes of Health Guidelines on the Use of Laboratory Animals and were approved by the Ethics Committee of Shanxi Provincial People Hospital. Wild-type (WT) mice (Male C57BL/6J, 8 weeks old) were utilized in this study. Mice were randomly assigned to two groups: normal diet (ND)-treated group; high-fat diet (HFD) (60% kcal fat, 20% kcal protein, 20% kcal carbohydrate, Catalogue No. D12492; Research Diets)-treated group. These two groups were fed with find or HFD for 24 weeks. Fasting blood glucose level and glucose tolerance were examined at 12 weeks of find or HFD. Type 2 diabetes are characterized by rising glucose (fasting blood glucose exceeded 12 mmol/L) and insulin resistance. Glucose tolerance: At the end of 12-hour fasting, blood glucose was measured via glucometer (Roche Accu-Chek^™^). Mice were then administrated with 1.5g/kg D-glucose via intraperitoneal injection, and blood glucose were measured at 30, 60, 120 and 180 minutes after glucose administration. Insulin tolerance: Mice were administrated with 0.5 units/kg Novolin R regular insulin via intraperitoneal injection after fasting for 4 h, and blood glucose were measured at 0, 30, 60, 90 and 120 minutes after glucose administration.

## Echocardiography

At 24 weeks of find or HFD feeding, mice were anaesthetized by continuous inhalation of 1–2% isoflurane. The anesthesia was determined sufficient if the mice did not respond to pinch with toothed pliers. After removing chest hairs using acoustic-coupled gel, serial M-mode echocardiographic images were obtained by GE Vivid 7 Pro System equipped with a 15-MHz linear transducer (General Electric Company, USA). Body temperature of mice was maintained between 36.5 °C – 37.2 °C during the entire experiments. Cardiac function of mice was assessed by left ventricular ejection fraction (LVEF) and the left ventricular fractional shortening (LVFS). We examined 6 mice in each group and measured each row for 3 times, and then took the average value.

## Histopathology

After performing the echocardiography at 24 weeks, the hearts were harvested, fixed with formalin and embedded in para n wax. Then, 5 μm serial sections were cut and placed on plus slides. The tissue sections were stained hematoxylin and eosin (H&E) to assess for the pathological changes in the cardiac tissues of each group. cardiac tissue sections were also stained using Masson trichrome kit (BA4079A, BASO, China). Staining images were captured with a Nikon Ni-SS microscope.

### Real-time quantitative PCR

Total RNA of cardiac tissue was extracted by Trizol kits (9108, TaKaRa, Japan), and then the cDNA was reversely transcribed by PrimeScriptTM RT Master Mix (Takara, Japan). Real-time PCR reactions were performed via the PrimeScript^™^ RT reagent Kit with gDNA Eraser (Takara, Japan). Primers were purchased from Sangon Biotech (Table 1). Relative quantities were assessed by the 2^–ΔΔCt^ method.

### Western blotting

Total proteins of cells or tissues were extracted by lysis buffer. BCA Protein Assay kit (AR0197, BOSTER, China) was used to measure protein concentration. Fifty μg total proteins per sample were subjected to gel electrophoresis (Bio-Rad, Shanghai, China), and transferred to polyvinylidene fluoride (PVDF) membranes. After blocking with 5% skimmed milk (room temperature, 1 h), the membranes were incubated with anti-mouse HSPB1 (1:200 dilution, #SC-13123, Santa Cruz Biotechnology, USA), anti-rabbit SLC40A1 (1:1000 dilution, A11156, ABclonal, China), anti-MGST1(1:500 dilution, ab131059, Abcam, UK), anti-rabbit GPX4 (1:1000 dilution, #52455, Cell Signaling Technology, USA), anti-rabbit SLC7A11 (1:1000 dilution, #98051, Cell Signaling Technology, USA) at 4°C overnight. Then the membranes were incubated with the secondary HRP-conjugated antibody (anti-rabbit antibody, anti-mouse antibody, 1:5000 dilution, Boster, China) at room temperature for 2h. Ultra-sensitive enhanced chemiluminescence detection kit (AR1191, BOSTER, China) was used to detect the bands of membrane, and the image was obtained via ChemiDoc MP Imaging System (Bio-Rad, USA). The bands of density were quantified by densitometry (Image Lab).

### Preparation of cardiac immune cell suspension and FCM

At 24 weeks, ND-fed and HFD-fed mice were both anesthetized, chest hairs were removed, open-chest surgery was performed to expose the heart, and heart was harvested for isolating single immne cells. Hearts were washed 3 times with PBS to remove blood and connective tissues. Heart tissues were digested with a digestive solution to obtain single immune cells. The composition of cardiac digestive solution: collagenase I (1.6 mg/mL, Gibco, USA) and DNase (0.2 mg/ml, MBI, USA) dissolved in PBS. The isolated immune cells were filtered using a 70-micron cell strainer. Cell suspension was stained with an antibody (BioLegend, USA) cocktail at 37 °C for 15 min and FCM was performed using a FCM system (BD Biosciences, USA). Immune cells were sorted as follows: CD4 + T cells, CD8 + T cells, Treg cells, B cells, NK cells, monocytes, macrophages, M1 cells, and M2 cells.

### Isolation of cardiomyocytes

Hearts were harvested as described above. Heart tissues were rinsed 3 times with PBS to remove blood and connective tissues. Ventricles was gently cut into 1-mm pieces and then were digested using collagenase II (Worthington, Lakewood, NJ). After achieving cellular dissociation, culture medium was added to terminate digestion. Cell suspension was filtered with a 100-μm filter, then the resuspended cells were preplated for 2 h to remove broblasts and to obtain isolated cardiomyocytes for culture.

### Cell culture and treatment

Cardiomyocytes isolated from the ND-fed and HFD-fed mice were cultured till 80% confluence. These cultured cells were cultured in normal DMEM medium (BOSTER, Shanghai, 25 mM D-glucose) or high glucose/high lipids (HGHL) DMEM medium (33 mM D-glucose and 500 μM palmitates) containing 10% fetal bovine serum (FBS) (BOSTER, Shanghai) and 1% penicillin/streptomycin/amphotericin B solution (BOSTER, Shanghai) in an incubator with 5% CO_2_ at 37 °C for 24 h.

To inhibit ferroptosis, the cardiomyocytes from ND-fed mice and HFD-fed mice were treated with 1 μM Fer-1 (Cat# A4371, APExBIO, USA) for 5 h. According to experimental design, the cells were divided into four groups (ND, HFD, ND + Fer-1, HFD + Fer-1), and ferroptosis was observed for all the cell groups.

### Cell counting kit-8 (CCK8) cell viability assay

Cardiomyocytes of the above two cell groups were both cultured in 96-well plates with normal or HGHL DMEM medium for 24 h and then were exposed to vehicle or Fer-1 for 5 h. Subsequently, 10 μL fresh CCK8 solution (C0038, Beyotime, China) was added to each well and incubated for 1 h at 5% CO_2_ and 37 °C. The absorbance values at 450 nm were detected using a microplate reader (Molecular Devices, USA).

### Statistical analysis

All statistical analysis were performed using the R version 4.0.3 and GraphPad Prism 8 software. Student’s *t*-test was used for comparison between two groups, and One-way ANOVA was used for multiple groups comparison. *P* < 0.05 was considered statistically significant. Each in vitro experiment was repeated at least three times.

## Results

### Bioinformatics reveals important role of FRGs in human DCM

**Figure 1:**
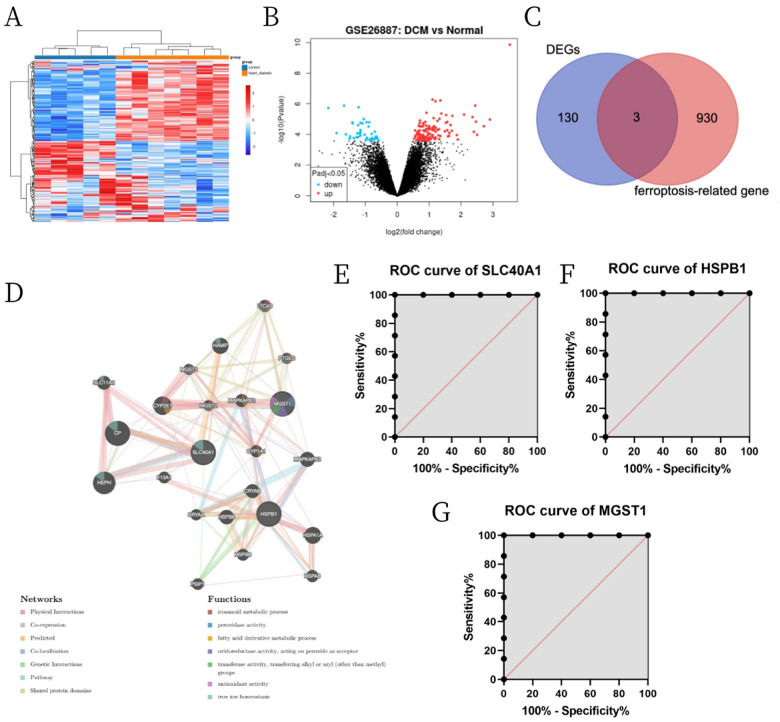
Identification of DE-FRGs and their role in DCM. (A) Volcano plot of DEGs. (B) Heatmaps of DEGs. (C) Venn diagram of DE-FRGs from GSE26887 and FerrDb. (D) PPI network of genes associated with three DE-FRGs. (E, F) ROC curve analysis revealing the diagnostic values of the three DE-FRGs in DCM.

A total of 133 DEGs were identified in GSE26887 database using the following criteria: |log (FC)| > 0.5 and adjusted *p*-value > 0.05. These DEGs included 33 upregulated and 100 downregulated genes (Fig. 1A, B). We also acquired a dataset that included 933 genes from the FerrDb. Venn diagram analysis of these two gene sets identified 3 DE-FRGs (Fig. 1C), including 2 downregulated genes (HSPB1, MGST1) and 1 upregulated gene (SLC40A1). The PPI network analysis of interacting genes with HSPB1, MGST1 and SLC40A1 were generated by GeneMANIA. The nodes in the network show that genes were related to three DE-FRGs in terms of physical interactions, co-expression, predicted, co-localization, genetic interactions, pathway, and shared protein domains. PPI network showed that three DE-FRGs significantly interacted with ceruloplasmin, hephaestin, cytochrome P450 family 2 subfamily E member 1, heat shock protein family A member 1A, hepcidin antimicrobial peptide, and other essential genes. Biological function analysis of these genes indicated the involvement of eicosanoid metabolic process, peroxidase activity, fatty acid derivative metabolic process, antioxidant activity, iron ion homeostasis etc. (Fig. 1D). ROC curve analysis was performed on the data of GSE26887 database to evaluate the diagnostic value of the three ferroptosis-related biomarkers in DCM. As a result, the accuracy of HSPB1, SLC40A1 and MGST1 in the diagnosis of DCM was all 100% in the GSE26887 (Fig. 1D–F).

**Figure 2:**
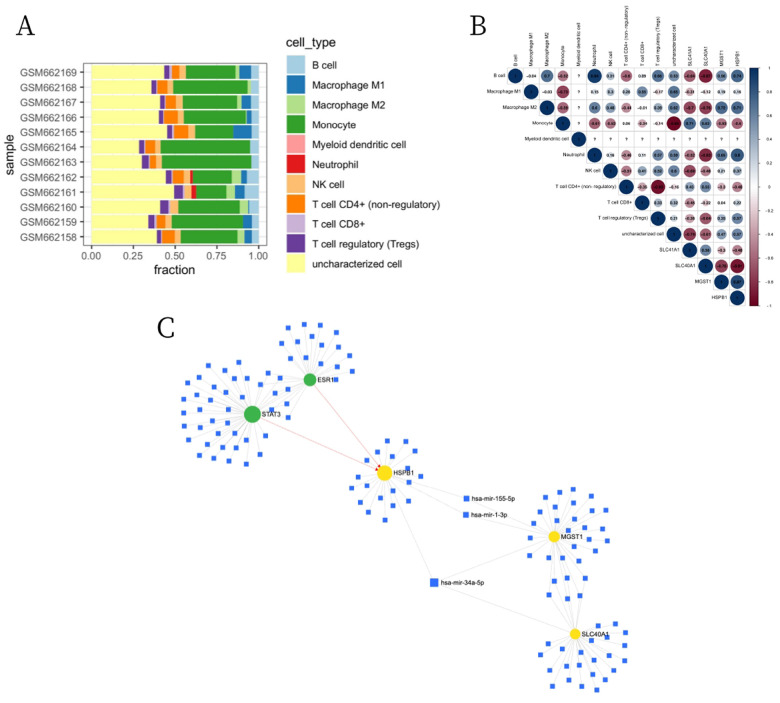
Immune infiltration analysis and miRNA regulatory network of DE-FRGs. (A) The enrichment fraction of 11 types of immune infiltrating cells in the DCM and normal samples. (B) Correlation heat map between 11 types of immune cells and DE-FRGs (blue indicates a positive correlation, red indicates a negative correlation, and the depth of the color indicates the degree of correlation). (C) Construction of the TF-miRNA-DE-FRGs network based on miRNet (blue indicates miRNA, green indicates TF, and yellow indicates DE-FRGs).

We further used the CIBERSORTx to assess the immune infiltration levels and proportions of 11 types of immune cells between normal patients and DCM patients (Fig. 2A). As shown in Fig. 2B, HSPB1 was positively correlated with B cell, macrophage M2, neutrophil, Treg cell and NK cell, but was negatively correlated with monocyte and CD4 + T cell (non-regulatory). SLC40A1 was positively correlated with monocyte and CD4 + T cell (non-regulatory), while was negatively correlated with B cell, macrophage M2, neutrophil and Treg cell. MGST1 was positively correlated with macrophage M2, neutrophil and B cell, but was negatively correlated with monocyte and CD4 + T cell (non-regulatory). In addition, the three DE-FRGs were uploaded to the miRNet online database to analyze their interactions with miRNA and transcription factors (TF) in humans. As shown in Fig. 2C, HSPB1, SLC40A1 and MGST1 were the targets of hsa-mir-34a-5p. Two TFs, STAT3 and ESR1, were closely related to HSPB1.

### HFD contributes to cardiac injury in mice

**Figure 3:**
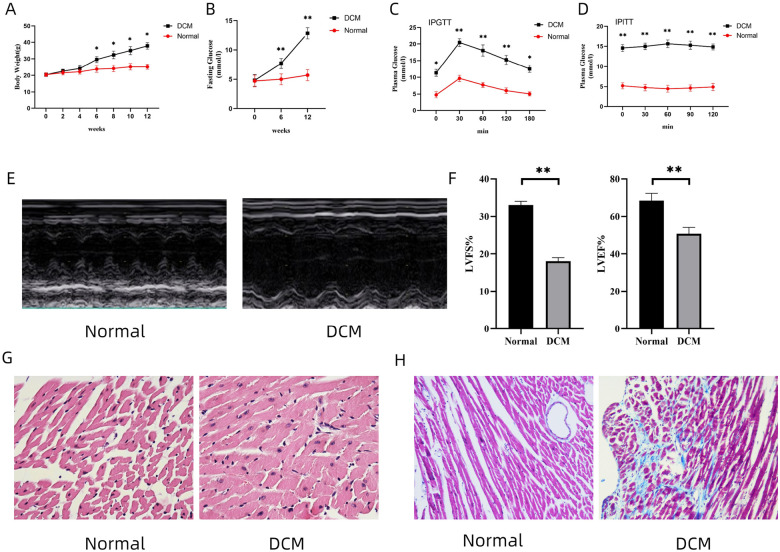
Manifestations of DCM and cardiac injury of mice in normal group and DCM group. (A, B) Body weight and fasting blood glucose of mice in both groups. n = 10 for each group. * *p*< 0.05, ** *p* < 0.01 vs. Normal. (C, D) Glucose tolerance and insulin resistance of mice at 12 weeks in both groups. Data are expressed as mean ± SD, n = 10 for each group. * *p* < 0.05, ** *p* < 0.01 vs. Normal. (E) Cardiac function of mice in different groups accessed by echocardiography. (F, H) left ventricle ejection fraction (LVEF) and left ventricle fraction shortening (LVFS) measured by echocardiography. Data are expressed as mean ± SD, n = 10 for each group. ** *p* < 0.01 vs. Normal. (G) Pathological changes of myocardium in mice (H&E, 20×). (H) Changes of cardiac fibrosis in mice (Masson, 20×). DCM, diabetic cardiomyopathy. LVEF, left ventricular ejection fraction. LVFS, left ventricular fraction shortening. H&E, hematoxylin and eosin.

To verify the immune infiltration in cardiac tissue and the expression of DE-FRGs in DCM, we prepared a DCM mouse model (HFD-fed for 24 weeks) [15]. At 12 weeks, compared with the ND-fed mice, the mice in the DCM group showed a significant increase in body weight (Fig. 3A). Meanwhile, the fasting blood glucose in the DCM group was higher than 12 mmol/L, and there were obvious symptoms of polydipsia, polyphagia and polyuria (Fig. 3B). The glucose tolerance and insulin resistance assays revealed that mice in the DCM group exhibited abnormal glucose tolerance and insulin resistance at 12 weeks (Fig. 3C, D). At 24 weeks, the cardiac functions of HFD-fed mice were significantly decreased, as evidenced by reduced LVEF and LVFS (Fig. 3E, F). Meanwhile, cardiac pathological changes were observed in the DCM group. H&E stains of mouse myocardium showed that, compared with the ND-fed group, the DCM group exhibited cardiac hypertrophy and disordered arrangement of cells in the myocardial tissue (Fig. 3G). Likewise, increased cardiac fibrosis was observed in the DCM group by Masson staining (Fig. 3H).

### Differential immune cell infiltration are found in the myocardium of DCM mice

**Figure 4:**
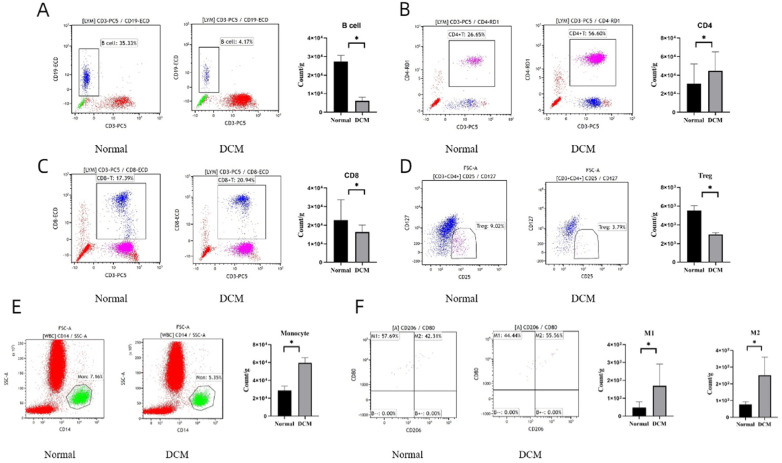
FCM results on the status of immune infiltration in Normal group and DCM group. (A-F) Count of each immune cell type per gram of the myocardial tissue (count/g) in Normal group and DCM group. Data are expressed as mean ± SD, n = 10 for each group. * *p* < 0.05 vs. Normal group.

To examine the immune infiltration in the heart of DCM mice, we prepared single immune cell suspension of myocardial tissue in the two groups (ND-fed group and HFD-fed group), and immune cell infiltration was analyzed using FCM (Fig. 4). An obvious alteration of immune cell composition was observed in the DCM group compared to the normal group, including CD4 + T cells, CD8 + T cells, Treg cells, B cells, monocytes, M1 and M2 macrophages (Fig. 4A–F). In the DCM group, B cells, CD8 + T cells and Treg cells showed a marked reduction (Fig. 4A, C, D). Conversely, the DCM group presented with increased levels of CD4 + T cells, monocytes, M1 cells and M2 cells (Fig. 4B, E, F). These results suggest a strong association of DCM with alterative infiltration of immune cells such as CD4 + T cells, CD8 + T cells, Treg cells, B cells, monocytes, M1 and M2 macrophages.

### Ferroptosis is involved in cardiac injury in mice with DCM

**Figure 5:**
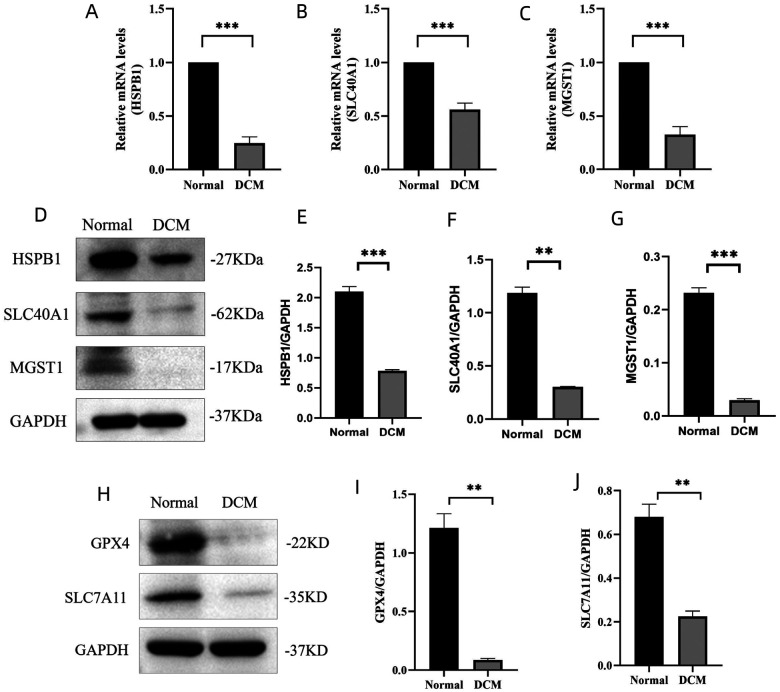
Ferroptosis in the progression of cardiac injury in DCM. (A-C) Transcription levels of HSPB1, SLC40A1 and MGST1 genes in Normal group and DCM group by accessed by quantitative real-time PCR. Data are expressed as means ± SD, n = 10 for each group. *** *p* < 0.001 vs. Normal. (D) Western blots showing the protein expression levels of HSPB1, SLC40A and MGST1 in both groups. (E-G) Quantification of the Western blot results. Data are expressed as mean ± SD, n = 10 for each group. ** *p*< 0.01, *** *p* < 0.001 vs. Normal. (H) Western blots of GPX4 and SLC7A11 in Normal group and DCM group. (I, J) Quantification of the Western blot results. Data are expressed as mean ± SD, n = 10 for each group. ** *p*< 0.01 vs. Normal. HSPB1, heat shock protein family B (small) member 1. SLC40A1, solute carrier family 40 member 1. MGST1, microsomal glutathione S-transferase 1. GPX4, glutathione peroxidase. SLC7A11, solute carrier family 7 member 11.

We examined the DE-FRGs expressions of HSPB1, SLC40A1 and MGST1 in mice cardiac tissues using quantitative PCR and Western blotting (Fig. 5). Compared to the normal group, the mRNA expression levels of HSPB1, SLC40A1 and MGST1 was remarkably decreased in the DCM group (Fig. 5A–C). Same results were obtained from the Western blots, the protein expressions of HSPB1, SLC40A1 and MGST1 in the DCM group was lower than that in normal group (Fig. 5D–G). Based on RNA and protein expressions, HSPB1 and MGST1 were both coincident with the result of bioinformatics analysis. It has been reported that HSPB1 is not only related to iron death, but also involved in regulating cardiac immune microenvironment in coronary artery disease [16]. MGST1 is considered as a key ferroptosis-related gene in type 2 diabetic mellitus [17]. Our finding indicated that the expression of HSPB1 and MGST1 in DCM group were downregulated compared with the normal group. To determine whether ferroptosis was involved in this process, we verified the expressions of ferroptosis related proteins (GPX4 and SLC7A11) by western blotting. Inactivation of GPX4 and SLC7A11 has been shown to promote ferroptosis. We showed that the cardiac expression levels of GPX4 and SLC7A11 in the DCM group was significantly lower than that in the normal group (Fig. 5H–J). Collectively, these results strongly suggest that ferroptosis is involved in the DCM of mice, and further, HSPB1 and MGST1 were likely linked to this process.

### Ferro ptosis is involved in HFD-induced cardiomyocyte damage

**Figure 6:**
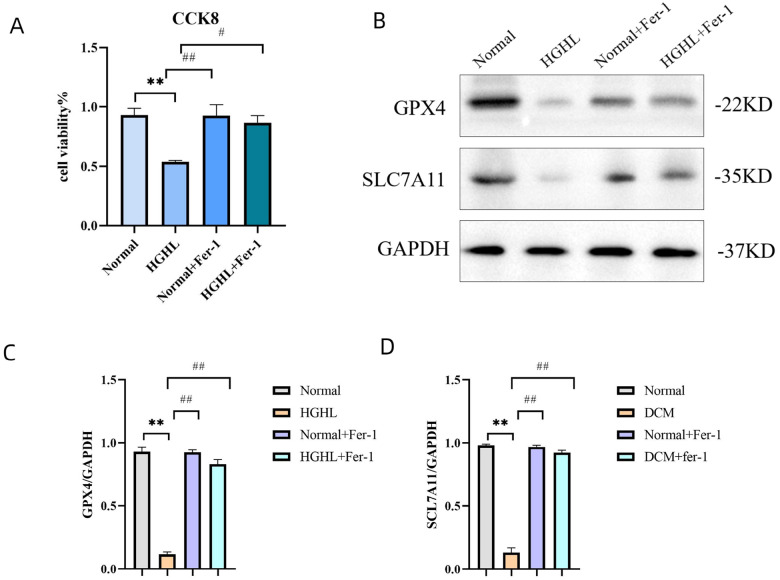
Ferroptosis involved in HGHL-induced injury in AC16 cells. (A) Viability of AC16 cells evaluated by CCK8 assay. AC16 cells were divided into four groups (Normal, HGHL, Normal + Fer-1, HGHL + Fer-1). Data are expressed as mean ± SD, n = 8 for each group. ** *p* < 0.01 vs. Normal; # *p* < 0.05 vs. HGHL; ## p< 0.01 vs. HGHL. (B) Western blots of GPX4 and SLC7A11 in the four groups (Normal, HGHL, Normal + Fer-1, HGHL + Fer-1). (C, D) Quantification of the Western blot results. Data are expressed as mean ± SD, n = 8 for each group. ** *p*< 0.01, vs. Normal; ## *p* < 0.01 vs. AC16, human cardiomyocyte cell line. HGHL, high glucose/high lipids. Fer-1, ferrostatin-1.

The above research showed that the ferroptosis plays a key role in DCM of mice. We next explored whether ferroptosis was induced in diabetic cardiomyocytes. To this end, we used isolated cardiomyocytes of mice to evaluate cell viability using CCK8 assay. Cardiomyocytes from the ND-fed and HFD-fed mice were both exposed Fer-1 or absence of Fer-1 for 5 h. Notably, the cell injury induced by HFD was significantly reversed by specific ferroptosis inhibitor Fer-1 as shown by the CCK8 assay (Fig. 6A), suggesting that ferroptosis plays a key role in HFD-induced cardiomyocyte injury. Subsequently, we detected GPX4 and SLC7A11 protein expressions in these four groups by western blotting. We found that the protein expression levels of GPX4 and SLC7A11 were reduced in the HFD group, but Fer-1 markedly reversed the low levels of GPX4 and SLC7A11 (Fig. 6B–D). Cell viability was decreased in cardiomyocytes from the HFD-fed mice, but was rescued by Fer-1. Moreover, the expressions of ferroptosis key suppressors GPX4 and SLC7A11 was significantly increased after Fer-1 treatment in the cardiomyocytes from HFD-fed mice. Collectively, these results strongly suggest that ferroptosis is an important cell death form in diabetic cardiomyocytes.

## Discussion

Diabetes mellitus (DM) often accompanied by severe cardiovascular complications (DCCs) which is the main cause of death in diabetic patients and DCM is the most common DCC in DM patients [18]. In recent years, many studies have suggested that ferroptosis plays a pathophysiological role in the development of cardiovascular diseases, including diabetic cardiomyopathy, myocardial infarction, heart failure, and myocardial ischemia-reperfusion injury [19, 20]. The pathogenesis of DCM includes oxidative stress and impaired antioxidant system under hyperglycemia [21]. As a result, imbalance of the antioxidant system often leads to excessive generation of reactive oxygen species (ROS), therefore the myocardium might undergo iron prolapse, apoptosis, inflammation, and fibrosis [22]. Recent studies indicate that nuclear factor erythroid 2-related factor 2 (NRF2) plays a pivotal role in maintaining cellular redox by regulating multiple antioxidants [23]. These regulatory genes include almost all genes encoding antioxidants involved in ferroptosis, such as glutathione regulatory genes, NADPH regeneration genes, lipid peroxidation genes, and iron regulatory genes [24, 25]. The search for new ferroptosis-related genes may reveal the pathogenesis of DCM and provide new ideas for research.

In the existing database, there are few DCM data on human specimens, and we screened 133 DEGs from the GSE26887 dataset. Three DE-FRGs were identified by interaction with the ferroptosis database. The diagnostic value of these three DE-FRGs in the GSE26887 dataset was verified by ROC curve, and the results showed that all of them had a large AUC. A larger AUC indicates a higher diagnostic value, but due to the small number of samples in this database, the significance of this result needs to be further determined. PPI network showed that three DE-FRGs significantly interacted with ceruloplasmin, hephaestin, cytochrome P450 family 2 subfamily E member 1, heat shock protein family A member 1A, hepcidin antimicrobial peptide and other essential genes. Biological function analysis showed that these genes were associated with eicosanoid metabolic process, peroxidase activity, fatty acid derivative metabolic process, antioxidant activity, iron ion homeostasis. Several studies have shown that these interacting genes are closely related to ferroptosis. Ceruloplasmin can suppresses ferroptosis by regulating iron homeostasis in hepatocellular carcinoma cells [26]. High expression of cytochrome P450 family 2 subfamily E member 1 can protect COS-7 cancer cells against ferroptosis. Our present study further demonstrated the role of these genes were associated with ferroptosis in DCM.

It has been shown that genes associated with ferroptosis can lead to different immune infiltrates in many diseases [27]. For the first time, we confirmed that all the three DE-FRGs (HSPB1, SLC40A1 and MGST1) were closely related to multiple immune cell infiltration in DCM. In addition, our analysis also identified HSPB1, SLC40A1 and MGST1 to be regulated by hsa-mir-34a-5p. These results also provide us with new research ideas for the mechanism of DCM, and further provide valuable insights into the intricate interplay between DE-TSRGs and immune cell infiltration in the context of acute myocardial infarction, potentially guiding future research into targeted therapies. These findings underscore the complexity of immune cell dynamics in the context of acute myocardial infarction and may provide valuable insights into the immunopathology of the condition.

To further validate the results of bioinformatics analysis, we established an animal model of DCM and confirmed its success through echocardiography and histopathology. Types and quantities of immune cells dramatically depend on different conditions of heart [28, 29]. The infiltration or depletion of immune cells are both involved in a variety of cardiovascular diseases [30, 31]. To verify the association between immune cells and DCM, FCM was used to detect immune cells in cardiac tissues. The results showed that CD8 + T cells, B cells and Treg cells were significant reduced in the DCM group, while CD4 + T cells, M1 cells, M2 cells and monocytes showed an obvious increase. These findings demonstrate the complexity of immune cells in the context of DCM and may provide potential guide into the immunopathology of this disease. Subsequently, we detected the cardiac expressions of HSPB1, SLC40A1 and MGST1 in mice, and both RT-qPCR and Western blotting results showed that the expressions of HSPB1 and MGST1 were consistent with the results of bioinformatics analysis. Compared with the normal group, the expressions of HSPB1 and MGST1 were significantly decreased in the DCM group. GSH is composed of glycine, amino acid cysteine, and glutamic acid. It is an effective lipid peroxidation scavenger and a key cofactor of GPX4, and the rate limiting precursor of glutathione synthesis is cysteine. Inactivation of the enzyme phospholipid hydroperoxide GPX4 and GSH depletion have been shown to promote ferroptosis [32]. SLC7A11 and 4F2HC constitute the cysteine-glutamate antiporter system, and most cells obtain cysteine from this system [33]. Thus, the GPX4-GSH-SLC7A11 axis plays a key role in the process of ferroptosis, and inhibition of GPX4 and SLC7A11 contributes to ferroptosis [34].

In this study, we detected the protein expressions of GPX4 and SLC7A11 in DCM mice, and the results showed that GPX4 and SLC7A11 proteins were significantly reduced, which is positively correlated with the expressions of HSPB1 and MGST1. Therefore, these results suggest that the reduction of HSPB1 and MGST1 is closely related to ferroptosis in DCM of mice, but further research is still needed. HSPB1 (also called mouse HSP25 or human HSP27), a member of the small heat shock proteins regulate iron homeostasis and prevent ferroptosis caused by high concentration of intracellular iron [23]. HSPB1 is an independent predictor of prognosis in patients with chronic heart failure, and its activation can also improve myocardial fiber arrangement and restore cardiac diastolic function [35, 36]. Here we showed that HSPB1 concentrations were lower in patients with type 1 diabetes than in non-diabetic healthy controls, and animal experiments showed that upregulation of HSPB1 is associated with neuronal protection [37, 38]. HSPB1 expression in cardiomyocytes is upregulated after myocardial infarction, and cardiomyocyte-specific HSPB1 deficiency promotes adverse remodeling after myocardial infarction, leading to cardiac dysfunction, cardiac rupture, and increased mortality [39]. At present, there are few in-depth studies on HSPB1 in diabetic cardiomyopathy and no relevant reports. MGST1 belongs to a superfamily named MAPEG (membrane-associated proteins in eicosanoid and glutathione metabolism), which is localized to the outer mitochondrial membrane and endoplasmic reticulum [40, 41]. It is an important mediator of inflammation and the main function is to reduce the lipid peroxide and protect intracellular membranes from oxidative stress [42]. Multiple studies have shown that MGST1 is closely related to the ferroptosis in cancer disease. In pancreatic cancer, MGST1 binds ALOX5 to inhibit lipid peroxide production during ferroptosis induction [43]. MGST1 can be used as a potential prognostic indicator and immunotherapy target based on iron ptosis in uterine corpus endometrial carcinoma [44]. Upregulated expression of MGST1 is associated with poor prognosis, enhancing the Wnt/β-catenin pathway via regulating AKT, and inhibiting ferroptosis in gastric carcinoma cells [45]. Overexpression of MGST1 induces gastric carcinoma cell proliferation by activating the Akt/GSK-3β signaling pathway. In type 2 diabetes, MGST1 was identified as the potential ferroptosis-related gene in islet beta cells dysfunction (IBCD) of type 2 diabetic mellitus (T2DM) by combing large patient samples of islet issue sequencing datasets and specificity of islet single-cell sequencing dataset [17]. At present, there are few studies on MGST1 in heart disease, and no study has been reported in diabetic cardiomyopathy. Our present study is also the first report.

Iron, an essential element for life, is closely associated with many biological processes, including nucleotide synthesis and repair and energy metabolism [46]. Iron overload is closely related to heart disease, for example, iron overload and injured function in heart have been observed in mice with deletion of cardiomyocyte-specific ferroportin (an iron exporter) gene [47]. Of note, the excessive cellular iron can react with oxidants through the Fenton reaction, thus promoting ferroptosis [48, 49]. Therefore, we used Fer-1 to explore whether ferroptosis was involved in HFD-induced cardiomyocyte damage. Our results showed that Fer-1 could effectively ameliorate the damage of diabetic cardiomyocytes and also increase the low expression of SLC7A11 and GPX4 in diabetic cardiomyocytes. This result suggests that ferroptosis is closely related with the cardiomyocyte injury induced by HFD-fed mice. Based on our bioinformatics analysis, 3 ferroptosis related genes (HSPB1, MGST1 and SLC40A1) were focused, and the expression levels of these genes were detected in the heart tissues of mice. We found that the changes in protein levels of HSPB1 and MGST1 was coincide with the results of bioinformatics analysis, and the expression of GPX4 and SLCA71in DCM group were significantly decreased. Our results indicate that ferroptosis-related proteins (HSPB1 and MGST1) are involved in DCM in mice. In order to confirm whether ferroptosis plays an important role in cells level, we pretreated isolated cardiomyocyte with Fer-1, and found that Fer-1 could protect against cardiomyocyte injury induced by HFD in mice and notably enhance the expressions of GPX4 and SLC7A11.

Our research also has several limitations. Firstly, the number of database samples we selected is relatively small, and the results of data analysis may have some randomness. Secondly, our bioinformatics analysis results show that DE-FRGs are closely related to immune infiltration and are regulated by miRNA. Further validation experiments in this area are needed. Thirdly, the role of Fer-1 is unclear in vivo. Hence, researches about improvement of myocardial damage after ferroptosis is blocked in DCM mice are necessary. In addition, we should knock down the levels of HSPB1 and MGST1 gene in DCM mice to study the status of myocardial damage, and further explore the signaling pathway of HSPB1 and MGST1 in DCM.

## Conclusion

We for the first time demonstrated that decreases of ferroptosis-related proteins of HSPB1 and MGST1 are involved in the process of DCM, and further identified that ferroptosis plays an important role in HFD-induced cardiomyocyte injury by bioinformatics analysis of human data, cell and animal experiments. HSPB1 and MGST1 are associated with multiple immune cells infiltration and are regulated by hsa-mir-34a-5p in DCM patients. In addition, immune cells of cardiac tissues changed remarkably in DCM. The study may provide a new insight in exploring the roles of ferroptosis and immunopathology for DCM research.

## Figures and Tables

**Table 1:** Primer sequence (5′–3′)

Gene name	Primer sequence (5 ′– 3 ′)
HSPB1	Forward: GCTCACAGTGAAGACCAAGGAAGG
	Reverse: GCACCGAGAGATGTAGCCATGTTC
MGST1	Forward: CGCACTGACGAGAAGGTGGAAC
	Reverse: GCCGATGCCGAGAAAGGGAAC
SLC40A1	Forward: TTGGTGACTGGGTGGATAAGAATGC
	Reverse: CGCAGAGGATGACGGACACATTC

## Data Availability

The datasets used and/or analyzed during the current study are available from the corresponding author on reasonable request.
